# Toward the Genetic Basis and Multiple QTLs of Kernel Hardness in Wheat

**DOI:** 10.3390/plants9121631

**Published:** 2020-11-24

**Authors:** Min Tu, Yin Li

**Affiliations:** Waksman Institute of Microbiology, Rutgers, The State University of New Jersey, 190 Frelinghuysen Road, Piscataway, NJ 08854, USA; mt751@waksman.rutgers.edu

**Keywords:** wheat, kernel hardness, linkage mapping, genotype–phenotype association, quantitative trait loci (QTL), *Puroindoline*

## Abstract

Kernel hardness is one of the most important single traits of wheat seed. It classifies wheat cultivars, determines milling quality and affects many end-use qualities. Starch granule surfaces, polar lipids, storage protein matrices and Puroindolines potentially form a four-way interaction that controls wheat kernel hardness. As a genetic factor, *Puroindoline* polymorphism explains over 60% of the variation in kernel hardness. However, genetic factors other than *Puroindolines* remain to be exploited. Over the past two decades, efforts using population genetics have been increasing, and numerous kernel hardness-associated quantitative trait loci (QTLs) have been identified on almost every chromosome in wheat. Here, we summarize the state of the art for mapping kernel hardness. We emphasize that these steps in progress have benefitted from (1) the standardized methods for measuring kernel hardness, (2) the use of the appropriate germplasm and mapping population, and (3) the improvements in genotyping methods. Recently, abundant genomic resources have become available in wheat and related *Triticeae* species, including the high-quality reference genomes and advanced genotyping technologies. Finally, we provide perspectives on future research directions that will enhance our understanding of kernel hardness through the identification of multiple QTLs and will address challenges involved in fine-tuning kernel hardness and, consequently, food properties.

## 1. Introduction

Wheat is one of the top three crops in global cereal production, providing about 28% of the world cereal production [1]. Wheat production involves the hexaploid common wheat (*Triticum aestivum* L.; 2n = 42, AABBDD) and the tetraploid durum wheat (*Triticum turgidum* ssp. *durum*; 2n = 28, AABB) [2]. Common wheat predominates in wheat production worldwide, while durum wheat contributes to ~7% of the production [3]. One major distinction between common wheat and durum wheat is the culinary applications of these species: durum wheat is milled to a specific type of flour, semolina, and is suitable for making pasta and spaghetti due to its high protein content, high gluten content and extremely hard grain texture [4], while common wheat can be used for making a variety of foods, from breads and noodles to cookies and cakes, depending on variations in kernel hardness, flour protein content and gluten content. Wheat kernel hardness is one of the most important single seed traits and partly determines the distinction between common wheat and durum wheat.

Kernel hardness is defined as the mechanical force used to crush wheat kernels. A major part of wheat kernel is the starchy endopserm, which is covered by multiple tissue layers, including outer pericarp, inner pericarp, testa, the hyaline layer (nucellar layer), and the aleurone cells [5]. As a wheat seed comprises these multiple tissue layers, kernel hardness is contributed by not only the starchy endosperm but also the outer layers, as well as the adhesion between endosperm and these layers [5,6]. The intrinsic biomechanical properties of the outer layers could be affected by: (1) cell structures and arrangements of each layer; (2) cell wall biochemical composition and the degree of cross-linking between different cell wall components; (3) cell wall thickness; and (4) the adhesion between different layers [5,6,7,8,9,10,11]. The biomechanical properties of the outer layers of wheat grain and the potential relationship between mechanical properties and kernel hardness are under studied compared to our knowledge in the hardness of starchy endosperm. Efforts have been made to measure the mechanical properties of isolated wheat grain layers using hysteresis tests, tensile stress and strain tests, uniaxial tension tests, traction tests, shear force tests, and puncture force tests, highlighting the differences in mechanical properties of the outer layers associated with wheat varieties, cell wall composition and other factors [5,6,7,8,9]. Pulsed laser ablation was established as a potential methodology for indirect measurement of the cohesion of wheat outer layers [10]. Another micromechanical device was developed to carry out peel tests for biological multilayered systems, like the wheat outer layers, and was used for determining the adhesion between wheat grain aleurone layer and nucellar layer [11]. Besides the studies on measuring micromechanical properties of wheat grain outer layers, methods have also been reported for in situ measurement of mechanical properties in the starchy endosperm [12,13]. Atomic force microscopy (AFM) nano-scratching approach has been employed to investigate local mechanical properties of the starchy endosperm, providing clear evidence for a higher hardness of starch compared to gluten [12]. More recently, Chichti et al. [13] reported an in situ investigation in the nano-mechanical properties of wheat starchy endosperm with this high-resolution method, and the results suggest the presence of a lipid layer at the starch-protein interface in the starchy endosperm of the soft wheat line, which is associated with the wild-type puroindolines with a potential lubricant role. By contrast, our understanding in endosperm hardness has been accumulated over several decades. Wheat endosperm has been long described by a model in which fillers (starch granules) are dispersed in a polymer (seed storage protein matrix) [4,14]. Based on this concept, the overall strength of the wheat endosperm is determined by its individual components (starch granules and protein matrix) and the adhesion between them. Furthermore, cereal chemistry studies have emphasized that it is the degree of adhesion between the storage protein matrix and starch granules rather than the hardness of the individual components in seeds that plays a major role in grain hardness [15,16,17,18]. The studies on biomechanical properties have confirmed that the interaction between starch granules and the protein matrix plays an important role in kernel texture [13]. More recently, the content of polar lipids on the starch granule surface has been linked to the breakdown of the amyloplast membrane, implying new insights into the interaction between polar lipids and puroindolines [19,20].

Kernel hardness largely determines the milling quality of wheat seeds, affects a set of physical and chemical seed properties, and influences a wide range of end-use quality parameters reviewed in [21,22,23,24,25]. Kernel hardness directly determines flour extraction or flour yield, and affects other milling parameters and flour properties, such as flour water absorption, break flour yield and the distribution of flour particle size [23,24,25,26,27,28,29]. Due to the significance of this trait in wheat end-use quality, the genetic basis of kernel hardness has drawn extensive research attentions for several decades. Kernel hardness, together with grain protein content (GPC), growing season and seed color, also involves in the classification of wheat cultivars in U.S. market, i.e., hard red winter wheat (HRW), hard red spring wheat (HRS), soft red winter wheat (SRW), hard white wheat (HW), and soft white wheat (SW).

The most commonly used methods for measuring kernel hardness are particle size index (PSI) [30], near-infrared reflectance spectroscopy (NIRS) [31] and single-kernel characterization system (SKCS) [32]. The former two methods depend on flour granularity. Particularly, NIRS-based hardness measurement indirectly estimates the particle size through the optical reflectance of flour samples [31]. Unlike the former two methods, the SKCS is a well-developed system for evaluating single-kernel properties. The SKCS records the crush-response profile of each single seed when the seed is crushed, providing much more information about the seed biomechanical properties. The SKCS quantifies kernel hardness in a continuous unit (namely hardness index, HI) ranging from 0–100, distinguishing soft- (HI < 40) and hard- kernels (HI > 60). Due to the suitability to measure a large amount of seed samples in a relatively short time, NIRS and SKCS were often used for collecting the phenotypes in the studies of mapping genetic loci regulating kernel hardness. Despite the wide use of NIRS and SKCS as the two major methods for measuring wheat kernel hardness, their relationship with another hardness-related measurement, vitreousness, has only been well discussed until recently using a set of near-isogenic lines (NILs) differing by *Pinb* alleles [28]. SKCS is affected by Pinb alleles and environmental factors. NIRS-based hardness exhibits a weaker correlation with SKCS hardness within a class of genetical hardness than when both genetically soft and hard genotypes are considered. Vitreousness has a weak relationship with NIRS-based hardness but a strong relationship with SKCS hardness index, with two different regressions for soft and hard genotypes [28]. Of note, the relationship between the different kernel hardness measurements goes beyond the scopes of this perspective, although the studies on this topic would help to explain, at least partly, the distinct kernel hardness-associated QTLs detected using NIRS or SKCS.

## 2. PINs Are the Major Causal Genes for Wheat Kernel Hardness

While the genetic control of wheat kernel hardness is polygenic, a major quantitative trait locus (QTL), the *Hardness* locus (*Ha*), was identified on the short arm of chromosome 5D decades ago [33,34,35] and accounts for ~60% of the phenotypic variations in kernel hardness [36,37]. In the 1980s, a group of proteins, namely Friabilin, was found in large amounts on the surface of water-washed starch granules in soft-kernel wheat lines, scarce in the hard-kernel wheat lines and was completely absent in durum wheat lines [38,39,40,41]. A series of biochemical separation [42,43,44,45,46] and amino acid sequencing studies [47,48,49,50,51] identified the two major proteins, Puroindoline a and b (PINA and PINB, respectively), and a minor protein, Grain Softness Protein-1 (GSP-1), composed of the friabilin (reviewed by Morris et al.) [52]. In the 1990s, several molecular biology studies further resolved the cDNA of *Pina* and *Pinb* [53,54]. Molecular genetics studies on the *Ha* locus revealed the key mutations in *Pina* and *Pinb* and their association with hard-kernel phenotype in common wheat lines [35,55,56,57]. The *Pina*, *Pinb*, and *GSP-1* genes, are closely linked and located on the *Ha* locus at chromosome 5DS [38,58]. These three genes encode a group of ~15-kDa cysteine-rich proteins that form a small clade in the phylogeny of wheat seed storage proteins, which belongs to the superfamily including a-amylase/trypsin inhibitors [53,59,60,61]. While some selected references have been mentioned in the above, the early studies on the identification and characterization of friabilin, Pina, Pinb and GSP-1 genes, and their effects on wheat kernel hardness have been systematically reviewed [21,22,52].

Durum wheat cultivars have very hard kernels (with a hardness index [HI] > 75) due to the lack of the D genome and, consequently, the *Ha* locus. In common wheat, the soft-kernel phenotype (with a HI < 40) requires the expression of both wildtype *Pina* and *Pinb*, whereas loss-of-function mutations in either *Pina* or *Pinb* lead to hard kernels [35,55] (reviewed by Bhave et al. [21,22]). The cause-and-effect relationship between *Pin* and wheat kernel hardness has been further proved using transgenic and complementation approaches [62,63,64]. Moreover, the effects of PINs on kernel softening have also been demonstrated in durum wheat, rice and maize [65,66,67]. In particular, the *Ha* locus has been transferred from common wheat into durum wheat variety Svevo using homoeologous recombination [68]. Subsequently, a series of soft-kernel durum wheat lines have been developed, with their grain characteristics, milling quality and food-processing qualities being comprehensively studied [69,70,71,72,73,74,75]. The development of soft kernel durum wheat lines and their significance have been reviewed [4,76]. Numerous studies have been focused on mining allelic variations and the genotype–phenotype association of *Pin*, resulting in the identification of 26 alleles of *Pina*, 33 alleles of *Pinb* and a few double null alleles [21,22,77,78,79,80,81,82,83,84,85,86,87]. Manipulation of the expression or genotypes of *Pin* not only modifies kernel hardness and milling properties in common and durum wheat lines [62,63,64,67,88,89,90,91,92], but also changes some parameters of the end-use quality and storage protein interaction [29,93,94,95]. Owing to its importance, research regarding *Pin* has been comprehensively reviewed by Bhave et al. [21,22] in aspects of its genetics, polymorphism identification and studies about its biological functions. Most recent research work on wheat kernel hardness and/or *Pin* has been updated in several reviews [23,24,25,27,96,97]. Detailed information on the genetics and biological functions of *Pin* and their effects on kernel hardness and end-use quality does not fall into the scope of the present review.

## 3. Dissecting the Genetic Loci Controlling Wheat Kernel Hardness

**Figure 1:**
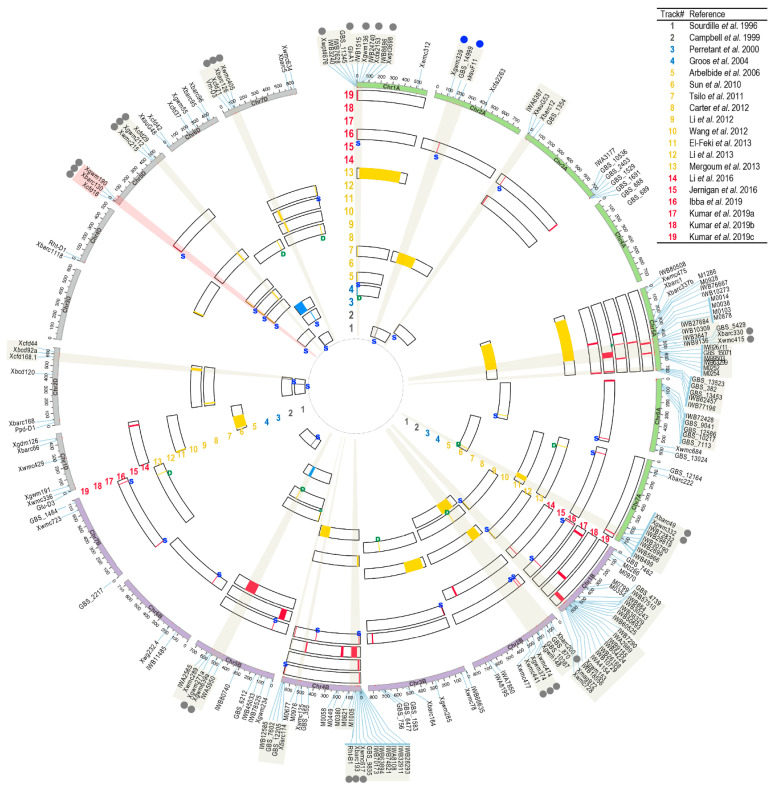
Projection of the QTL regions and genetic markers associated with wheat kernel hardness on the reference genome of common wheat Chinese Spring (CS42, RefSeq v1.0) [2]. The QTL regions associated with kernel hardness were retrieved from nineteen QTL mapping studies reviewed in this article [36,37,98,99,100,101,102,103,104,105,106,107,108,109,110,111,112,113,114,115,116,117,118]. Detailed information about these QTL-mapping studies and their projected QTLs or markers are provided in Table 1 and Supplementary Table S1. The left and right boundaries of a QTL are defined by two genetic markers according to the corresponding study. In some cases, only the left or right marker can be accurately located on the reference genome of CS42, leaving only one kernel hardness-associated marker projected onto the map. If both the left and right boundary markers can be located on the genome, the QTL region is indicated using a green letter “D” (meaning “double markers”). If only the left or right boundary marker can be located on the genome, the QTL/single marker is indicated using a blue letter “S” (meaning “single marker”). The out-most track denotes the wheat genome assembly. The A, B and D genomes are colored in green, purple and gray, respectively. The outer to inner tracks represent the 19 QTL-mapping studies, labeled using the first author and year of publication. For each study (track), only the chromosome on which a kernel hardness-associated QTL region could be located is shown, with the QTL regions or markers highlighted in colors (i.e., gray, blue, yellow and red). The colors used to highlight the QTL regions, i.e., gray, blue, yellow and red, denote the main types of markers used in each QTL mapping study, namely, RFLP, RFLP + SSR, SSR + DArT and SNP, respectively (Table S1). All of the markers used for QTL projection are labeled outside the wheat genome track.

Generally, the mutations of *Pin* determine the major kernel-hardness class of wheat. However, continuous variations in kernel hardness have been often observed within each class among natural populations or bi-parental segregating populations, implying that many QTLs with small effects remain to be discovered. Indeed, population genetic studies have confirmed that kernel hardness is a quantitatively inherited trait (heritability > 0.7) and that polymorphism in Pin can only explain over 60% of the trait’s variation [36,37]. Since the 1990s, many efforts have been made to uncover the genetic basis of wheat kernel hardness. On the one hand, several studies have confirmed that the *Ha* locus on chr. 5DS is the the major genetic factor [36,37,98,99,100]. On the other hand, minor QTLs with smaller effects have been identified in almost all chromosomes of wheat. One major challenge for mapping kernel hardness was that the QTLs detected in early studies had limited resolution power, and each QTL could cover wide genomic regions due to the limited number of traditional genetic markers, such as restriction fragment length polymorphisms (RFLPs), amplified fragment length polymorphisms (AFLPs) and simple sequence repeats (SSRs), and, consequently, low-density linkage maps [37,98,99,101]. Another challenge was that the effects of polymorphic Pin could hinder the identification of other QTLs with minor effects in bi-parental mapping populations using soft-kernel and hard-kernel lines as the parents. These limitations have been substantially resolved over the past ten years. Here, an effort is made to review the literature on QTL mapping of wheat kernel hardness. Of the twenty-one reviewed papers, eighteen studies used bi-parental linkage mapping, while two used association mapping (Table 1; Tables S1 and S2; Figure 1 and Figure S1). The progress in kernel-hardness QTLs is summarized.

Sourdille et al. [36] mapped kernel hardness using a recombinant inbred line (RIL) population with RFLP markers, and they identified the major QTL on 5DS, the *Ha* locus, as well as a number of minor QTL regions, including four regions on 2A, 2D, 5B and 6D and three regions (on 5A, 6D and 7A) with interaction effects. Campbell et al. [37] discovered two kernel hardness-associated QTLs using a recombinant inbred line (RIL) population with RFLP markers, including the *Ha* locus and another minor genetic factor on chr. 3AS. Combining RFLP, AFLP, SSR, and functional markers, Perretant et al. [98] detected two minor QTL regions on chromosomes 1A and 6D. To eliminate the major genetic effects of the *Ha* locus and to reveal other minor factors more effectively, Groos et al. [101] used a RIL population from a cross between two hard wheat cultivars to map kernel hardness, which was determined by near-infrared spectroscopy (NIRS; Hard_NIR_) and a single kernel characterization system (SKCS; Hard_SKCS_), respectively. In this study, eleven QTLs associated with each of the Hard_NIR_ and Hard_SKCS_ measures were identified, with individual QTLs explaining from ~5% to ~27% of the phenotypic variation [101]. Another QTL mapping study that used a bi-parental RIL population with RFLP and SSR markers confirmed the *Ha* locus as the major genetic factor and identified a minor QTL on chr. 1B [102]. A QTL mapping study using multi-parental elite inbreds from the Limagrain Genetics wheat breeding program detected two chromosomal regions associated with kernel hardness, one on chr. 1AL and another on chr. 5DS (the *Ha* locus) [99]. Another QTL mapping study used a RIL population from a cross between soft and hard wheat cultivars and identified two QTLs for NIRS-based kernel hardness and four QTLs for SKCS-based kernel hardness, highlighting the significant role of the *Ha* locus and the different genetic factors associated with the two types of kernel hardness measures [100].

More recently, mapping populations developed from only soft-kernel or hard-kernel parents were reported in several studies, which helped to improve the efficiency for detecting minor QTLs associated with kernel hardness. A QTL mapping study using a RIL population generated by two hard red spring wheat lines and several hundreds of SSR and diversity array technology (DArT) markers revealed additional QTLs associated with kernel hardness besides the *Ha* locus [103,104]. A study that aimed to identify the underlying genetic factors controlling the “extra-soft” kernels found four QTL regions based on the results collected over multiple years and from multiple environments [105]. Li et al. [106] mapped the genetic factors of kernel hardness using two related bi-parental, hard-kernel RIL populations and identified three and five kernel hardness-associated QTLs in each population, respectively, across multiple environments. None of these QTLs overlaps with the *Ha* locus on chr. 5DS. Kernel hardness-associated QTLs were also detected on wheat chromosomes 2B, 2D, 4B and 6A in a soft-kernel RIL population [107]. El Feki et al. [108] investigated the genotype–phenotype linkage for a bi-parental, hard-kernel double haploid (DH) line population and detected six QTLs associated with kernel hardness, two of which were significant across multiple environments. Li et al. [109] employed a RIL population with a bi-modal hardness index distribution and revealed one major QTL on chromosome 7D that explained 33% of the phenotypic variation. In another report, four kernel hardness-associated QTLs were found to explain large phenotypic variations (from 22% to 26%) in a bi-parental, hard-kernel RIL population [110].

Recently, advanced technologies, such as wheat single-nucleotide polymorphism (SNP) arrays, Genotyping-by-Sequencing (GBS) and genome-wide association analysis (GWAS), have been applied to improve the efficiency and resolution power for detecting QTL regions associated with kernel hardness. Li et al. [111] obtained ~600 SNP markers with the Infinium iSelect wheat genotyping SNP array and identified two and five QTLs for NIRS-based and SKCS-based kernel hardness, respectively. Boehm Jr. et al. [112] identified over 600 GBS-derived SNPs and detected four QTLs in a bi-parental, hard-kernel RIL population on chromosomes 1AS, 1BS, 5BL and 7BS. In contrast to Boehm Jr. et al. [112], who identified the genetic factors for hard-kernel variations, Kumar et al. [113] constructed a QTL mapping population using a cross between the soft and extra-soft wheat cultivars. In this study, over 1400 SNP markers were identified using the 90K Infinium iSelect SNP array, and the high-density genetic map facilitated the identification of ten soft-kernel-associated QTLs on chromosomes 5AL, 7AS, 1BS and 4BS. In further generations of this RIL population, the QTLs on chromosome 4BS, 1BS and 5AL were validated. Additional kompetitive allele-specific PCR (KASP) and SNP markers were developed to help marker assisted breeding for super-soft wheat varieties [114]. More recently, the number of usable SNP markers for genetic mapping has been further improved. In a QTL mapping study of durum wheat, Ibba et al. obtained ~8500 dominant GBS-SNP markers and discovered two major QTLs on chromosomes 3A and 6A, as well as 18 significant GBS-SNP signals, implying that the complexity of the genetic control of kernel hardness extends beyond the *Ha* locus [115]. Kumar et al. generated a high-density linkage map using over 10,000 SNP markers and detected seven QLT regions linked to kernel hardness across multiple environments [116].

In addition to linkage mapping studies, the GWAS approach has been used in wheat for identifying kernel hardness-associated SNP signals, too. Two SNP signals, one on chr. 2A and another linked with the *Ha* locus, were identified using a GWAS population that comprised 94 elite lines with ~1200 DArT and SSR markers [117]. In a large-scale GWAS analysis of end-use quality traits, 462 soft-kernel wheat lines were genotyped with over 15,000 high-quality SNPs, and their historical phenotyping data were used. The marker–trait association revealed five QTLs of kernel hardness, including one on chr. 5A [118].

In summary, previous studies have identified numerous kernel hardness-associated genetic loci other than the well-studied *Ha* locus. Previously, the genomic or genetic resources for summarizing and projecting these genetic loci in a single genome or a consensus genetic map were not available. Recently, contiguous and high-quality genome assemblies have been produced for several *Triticeae* species, including common wheat (*Triticum aestivum*), durum wheat (*Triticum turgidum* spp. *durum*), wild emmer wheat (*Triticum turgidum* spp. *dicoccoides*) and the progenitors of the wheat A and D genomes, *Triticum urartu* and *Aegilops tauschii*, respectively [119,120,121,122,123]. These *Triticeae* genomes have greatly improved the characterization of several gene families and superfamilies, for example, wheat seed storage proteins, avenin-like proteins, MADS-box genes, and *Pin* and *Pinb2* genes [60,61,124,125]. In particular, the common wheat genome (International Wheat Genome Sequencing Consortium (IWGSC) RefSeq v1.0 for the cultivar Chinese Spring (CS42)) allows the accurate assignment of the genomic positions of the majority of the genetic markers that have been associated with kernel hardness QTLs, including SSR, AFLP, DArT and SNP markers (Table S1). The information on the markers was collected from a number of sources. First, the chromosomal locations, primers/probes of the kernel hardness-associated SSR, AFLP and DArT markers were retrieved from GrainGenes (https://wheat.pw.usda.gov/GG3) and were used to determine the genomic positions of the markers in the wheat genome of CS v1 using the BLAST service provided by the Tritieace multi-omics center (http://202.194.139.32/outsitelink.html). Second, DArT probes were retrieved from diversityarrays.com and previous references [126,127] and used to determine the genomic positions of DArT markers. Third, SNPs from wheat genotyping SNP arrays were obtained, and their genomic positions were determined using SNP-detecting probes (USDA wheat SNP database: https://wheat.pw.usda.gov/GG3/node/147.) [127,128]. Fourth, the genomic positions of the GBS-derived SNPs were obtained according to previous studies [112,115]. Based on the genomic positions of these markers, we projected the kernel hardness-associated QTLs on the IWGSC wheat genome v1 with Tbtools (Figure 1) [129]. The kernel hardness QTLs were identified on almost every chromosome of wheat, except for chr. 4A and 3D. Multiple previous studies consistently identified the *Ha* locus on chr. 5DS (highlighted in red in Figure 1). Moreover, by comparing the genomic positions of these QTLs, we observed genomic regions with overlapped or co-localized QTL regions that were detected in different studies using different mapping populations (highlighted in grey in Figure 1).

While the majority of the kernel hardness-related markers can be projected onto the wheat genome assembly, the genomic positions are still lacking or cannot be accurately assigned for some genetic markers. In such cases, projection onto a genetic map serves as a complementary approach. Owing to the increasing number of different types of genetic markers, a high-density consensus genetic map that compiles 7352 markers has been reported [130] and is used here for the projection of kernel hardness-associated QTLs (Figure S1, Table S2). While the QTLs subjected to fewer studies can be located on the consensus genetic map, both QTL locations on the genetic and genome maps are consistent. Several QTLs on chromosomes 1A, 2A, 5A, 2B, 4B, 5B, 5D and 7D can be projected onto similar chromosomal segments on both maps (the associated markers that are highlighted using dots in Figure 1 and Figure S1). A total of ninety-nine QLT were projected on the bread wheat reference genome, while fifty-four QTL were projected on the consensus genetic map. For the QTL summarized here, some has only one marker that could be projected on the genome or the genetic map, and some has been reported with only one associated marker, with some single trait-associated marker identified using the GWAS approach or single marker association approach (SMA) [115]. The QTL size distribution are plotted for those QTL of which two markers were reported and could be projected on either the genome or genetic map (Figure S2). Among the forty-five QTL used for physical size estimation, most of the QLT range from 53.2 Mb to 7.4 Mb in size. Among the twenty-one QTL used for genetic distance estimation, the majority ranges from 37.7 cM to 10.1 cM. For those single markers associated with kernel hardness in the GWAS or SMA analyses, the causal gene could likely reside within the local LD decay distance. Wheat is a species with a very large genome and a long LD decay distance. The average genome-wide LD block size was 4.2 Mb, and the local LD decay distances varied between sub-genomes and chromosomes, with many large LD blocks detectd on the A sub-genome, spanning a couple of million basepair [116]. The large LD block sizes reported in the wheat population studies are in line with the sizes of kernel-hardness QTL summarized here [131,132].

While many QTL have been reported to be associated with kernel hardness, the candidate genes or causal genes are still unknown. Moreover, many QTL were only detected in a single study. One explanation for this phenomenon could be that kernel hardness is also affected by environments [25,28,97]. For example, Oury et al. [28] dissected the genetic and environmental contributions to several kernel hardness-related measurements, including SKCS-HI, NIRS hardness and vitreousness, demonstrating that environment factors affect all of the three kernel-hardness measurements. Another example highlighting environment-related changes in kernel physical properties is that the degree of vitreousness, an optical characteristic related to endosperm porosity, were dramatically changed in puroindoline near-isogenic lines [133]. Another explanation for the many QTL detected in a single study may be the phenotyping method. The wheat milling process can be divided into two phases: the separation between bran layers and endosperm, and the size reduction of endosperm particles. It has been shown that NIRS-based hardness is related to both the bran-endosperm separation and endosperm reduction, whereas SKCS-based hardness and vitreousness are mainly involved in the structures of starchy endosperm [134]. These results indicate that the different methods for quantifying kernel hardness are complementary, reflecting various physical or biomechanical properties from different seed tissues. In light with the kernel-hardness QTL studies and the new advances on the factors involved in the grain structure, multi-environment GWAS analysis using complementary approaches to collect kernel-hardness phenotypes would be of vital importance to portrait the comprehensive genetic architectures of wheat kernel hardness, which are attributed by different layers of seed tissues with intrinsically distinct mechanical properties.

Table 1 shows brief information regarding the studies reviewed here, in which kernel hardness QTLs or associated SNPs were identified and can be projected onto the common wheat reference genome or consensus genetic map. In particular, for the study by Kumar et al., 2019b [114], the size of the genetic map is noted as “NA” (Not applicable), because this study is a confirmatory study of Kumar et al., 2019a [113]. Sixteen KASP markers and three SSR markers around the previously identified QTLs (Kumar et al., 2019a [113]) were selected and used for single marker-association analysis (SMA). NM = not mentioned.

## 4. Technological Improvements Benefit the Mapping of Kernel Hardness

Much progress has been achieved in understanding the genetic basis of wheat kernel hardness over the past two decades. In particular, improvement in the resolution power of wheat QTL mapping has helped to unveil many QTL regions with minor effects on kernel hardness in different populations.

**Figure 2:**
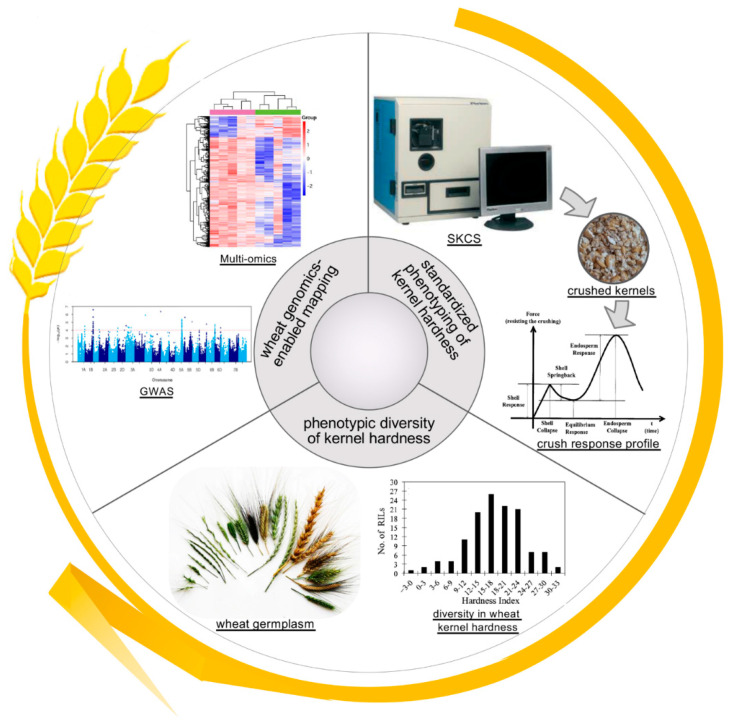
Advancement in phenotyping, germplasm and genotyping technologies has facilitated our understanding of the genetic basis of kernel hardness in wheat. An essential first step toward unveiling the genetic basis and multiple QTLs of kernel hardness is to establish a robust phenotyping method. SKCS provides a standardized measurement of kernel hardness and considers interfering factors, including kernel weight, diameter and water content, and individual variations between kernels. Secondly, appropriate germplasm or mapping populations are required to identify the multiple QTLs associated with wheat kernel hardness, because the *Ha* locus on chr. 5DS plays a major role in kernel hardness and could complicate the detection of other minor QTLs. Subsequently, recent advancements in wheat genomics and population genetics have provided the tools and resources for high-resolution phenotype–genotype association: (1) a large number of SNPs is necessary for high-density linkage mapping or association analysis; (2) a contiguous, high-quality reference genome of wheat is necessary for linkage mapping, GWAS analysis and candidate gene identification; (3) multi-omics data on wheat are increasingly accumulating, providing additional resources for narrowing down and prioritizing candidate genes of kernel hardness.

The recent advances in mapping wheat kernel hardness have benefited from improvements from three technological aspects (Figure 2): (1) a standardized phenotyping method of wheat kernel hardness; (2) harnessing of the phenotypic diversity of wheat kernel texture, and the creation and utilization of the appropriate population for kernel-hardness mapping; and (3) the availability of powerful genomic resources in wheat.

First, QTL mapping or GWAS analysis of kernel hardness requires phenotype measurement of several hundreds of samples over multiple years and/or from multiple environments, for which an instrument-based or semi-automated phenotyping method is desired to minimize the variations that arise from manual operation and other interfering factors. Currently, two methods for measuring kernel hardness are widely used, i.e., near-infrared spectroscopy (NIRS) and the single kernel characterization system (SKCS). The results of both methods are largely correlated, although the extent of the correlation varies between studies, with a correlation coefficient (*r*) ranging from 0.53 to 0.81 [101,111,112]. Such differences between NIRS-based kernel hardness (Hard_NIR_) and SKCS-based kernel hardness (Hard_SKCS_) reflect in the largely non-overlapped QTL regions associated with Hard_NIR_ or Hard_SKCS_ [28,100,101,111,112,135]. In particular, SKCS is likely the only commercially available instrument that directly measures the compressive strength of a kernel and was developed to meet the industrial needs for an objective and reliable classification method of wheat varieties [136,137,138,139]. SKCS has several advantages in measuring cereal kernel hardness: (1) it offers a simple, rapid (a few minutes per sample) and direct measurement of kernel strength; (2) it considers other interfering factors, such as kernel diameter, kernel weight and water content; (3) it considers the individual variations in these kernel parameters and can measure kernel parameters for a sample of hundreds of kernels; (4) it records the kernel strength as a crush-response profile, which separates the crush responses of seed shell and endosperm and provides detailed information on kernel hardness. Indeed, SKCS-based measurement has been widely used for mapping wheat kernel hardness and was chosen by fifteen out of the twenty-one studies reviewed here.

Second, identification and utilization of wheat germplasm and populations suitable for mapping kernel hardness are a critical step toward unveiling multiple QTLs. Since *Pina* and *Pinb* on the *Ha* locus were identified as the major causal genes of kernel hardness in the 1990s [52], several groups chose to develop mapping populations using only soft-kernel or hard-kernel lines as the parents [101,104,105,106,107,108,109,110,112,113,114,116,117]. Such a strategy could eliminate the dominant effects of the *Ha* locus on kernel hardness in the population. When a bi-parental population was made by a cross between two hard-kernel or soft-kernel wheat cultivars, a normal distribution of kernel hardness within the hard-kernel or soft-kernel class was observed in many cases, suggesting that a number of genetic loci with small effects are involved in the regulation of kernel hardness. In a few cases, a bi-modal distribution of kernel hardness was detected, indicating that another major gene was affecting kernel hardness [107]. In addition, the identification of special wheat lines with extreme hardness indices and their utilization in mapping populations have played important roles in enhancing our knowledge of kernel hardness. In contrast to normal soft-kernel lines with HIs of ~20, super-soft lines with HIs of less than 5 were used to discover novel QTLs for kernel hardness [105,113,114,117].

Third, the application of genomic technologies (i.e., GBS and SNP arrays) increases the resolution power for mapping kernel hardness QTLs in wheat (Figure 2). GBS-derived and SNP-array-based SNPs have increased the number of polymorphic markers (SNPs) from several hundred to several thousand [111,112,113,114]. This increase in the number of markers, together with appropriate mapping populations, has led to the successful identification of many QTL regions with small effects on kernel hardness. Moreover, a few studies have used the GWAS approach to localize kernel hardness QTLs [117,118]. Compared with linkage mapping, GWAS has three major advantages: (1) GWAS does not require the development of mapping populations, saving time and resources [140]; (2) GWAS can detect more alleles and has higher resolution power for detecting trait-associated signals due to the broader genetic diversity of GWAS panels and the higher number of SNP markers [141]; (3) the trait-associated SNPs identified in a GWAS study can be used for genomic selection or improving genetic gains in breeding.

## 5. Conclusions and Future Perspective

Recently, abundant genomic resources have become available and cover several aspects of multi-omics (Figure 2). First, the release of several high-quality *Triticeae* genomes has laid the foundation for performing GWAS analysis, connecting physical and genetic maps and identifying candidate genes within QTL regions [2,119,120,121,122,123]. Second, a series of high-density SNP genotyping arrays provide versatile scenarios for wheat genotyping for varying costs and applications [126,127,128,142,143,144]. These wheat SNP-genotyping arrays have been comprehensively compared and reviewed [145]. More importantly, these SNP arrays increase the number of SNPs available for QTL mapping from several thousand to tens or hundreds of thousand at affordable costs, allowing the accurate mapping of minor QTLs [146,147,148]. Third, as the complements to array-based genotyping, datasets of wheat whole-genome resequencing [149,150,151], representative sequencing (such as GBS) [131,132] and exome sequencing [152,153] have been recently published, enriching the repertoire of wheat genomic resources. These new resources are powerful not only for association mapping but also for identifying potential functional changes in genes and regions subjected to domestication and/or selection. Fourth, transcriptomic resources of wheat are increasingly accumulating [154,155,156]. Other multi-omic resources are emerging, including epigenomics [157,158,159].

With these new multi-omics resources of wheat in hand, further studies of kernel hardness should focus on the following objectives: (1) comprehensively reveal the genetic architectures of kernel hardness and QTL regions other than the *Ha* locus on chr. 5DS using soft-kernel or hard-kernel wheat populations; (2) fine-map kernel-hardness causal genes with high-throughput markers derived from NGS technologies, and prioritize candidate genes within kernel hardness QTLs with multi-omics datasets; and (3) functionally characterize candidate genes with the emerging wheat mutant resources [160,161]. A major bottleneck to illustrating the genetic basis of kernel hardness is that functional evidence of prioritized candidate genes within kernel hardness QTLs is scarce, while many QTL regions have been reported. In one example, Tsilo et al. identified a QTL linked to chr. 5DS using a bi-parental population in which the two parents both had an identical Pin genotype and the hard-kernel phenotype, indicating that another gene, likely closely linked to the *Ha* locus, may be responsible for the phenotypic variation [89]. Recently, a transgenic study of GSP on the *Ha* locus showed that regulation of GSP can have a small effect on grain hardness, coinciding with the minor QTL detected on chr. 5DS [161].

In conclusion, continuous efforts in mapping wheat kernel hardness have become fruitful over the past two decades, and numerous QILs have been identified, including some QTLs that were detected in multiple independent studies. In the future, harnessing the genetic diversity and integrating new genomic resources and technologies will be the key to fully uncovering the genetic architecture of kernel hardness and the underlying causal genes in wheat. New knowledge of the genetics of kernel hardness will enhance our understanding of the mechanical strength and structure of cereal seeds, facilitate marker-assisted selection for this trait, improve the genetic gains of breeding and help to develop new wheat germplasm with fine-modulated kernel hardness and end-use properties.

## Figures and Tables

**Table 1:** Summary of QTL studies included in this analysis.

References	Track# Genet Map ^1^	Track# Genom Map ^2^	Description of the Mapping Population	Type of Pop. ^3^	Pop. Size ^4^	Genetic Map Size ^5^	Marker Type	No. QTL	Projected No. QTL Genetic Map	Projected No. QTL Genomic Map	5DS *Ha* Locus
Sourdille et al., 1996 [37]	1	1	W-7984 X Opata85 Soft X Hard	RIL	86	NM	RFLP	5	5	3	Yes
Campbell et al., 1999 [38]	2	2	NY18 X CC Soft X Hard	RIL	78	1806	RFLP	4	1	4	Yes
Perretant et al., 2000 [100]	3	3	Courtot X Chinese Spring (CS) Hard X Soft	DH	169	~2900	RFLP, AFLP, SSR, functional markers	3	3	1	Yes
Groos et al., 2004 [103]	4	4	Renan X Recital Hard X Hard	RIL	165	2722	RFLP, AFLP, SSR,	5 ^6^	3	3	
Arbelbide et al., 2006 [101]	5	5	158 F2 crosses made from 80 parental lines, and SSD to generate 373 RILs	RIL	373	NM	SSR, functional markers	2	2	2	Yes
Sun et al., 2010 [102]	6	6	Ning7840 X Clark Hard X Soft	RIL	132	2203	AFLP, SSR	2; 4; ^7^	4	5	Yes
Tsilo et al., 2011 [106]	7	7	MN98550 X MN99394 Hard X Hard	RIL	139	2505	SSR, DArT, functional markers	6	6	6	Yes
Wang et al., 2012 [107]	10	10	OS9A X Q36 Soft X Extra Soft	RIL	164	1821	SSR, DArT, functional markers	4	4	4	
Li et al., 2012 [108]	9	9	Weimai 8 X Jimai20 Weimai 8 X Yangnong19 All three parents are hard kernel	RIL	485-WJ; 229-WY;	2855-WJ; 3010-WY;	SSR, STS, SRAP, RAPD, functional markers	3; 5; ^8^	4	6	
Carter et al., 2012 [109]	8	8	Louise X Penawawa Soft X soft	RIL	188	NM	SSR, SNP (only 1), functional marker	4	4	4	
El-Feki et al., 2013 [110]	11	11	CO940610 X Platte Hard X Hard	DH	185	2117	SSR, STS, DArT	6	6	5	
Li et al., 2013 [111]	12	12	R146 x R97 Hard X Hard	RIL	103	NM	SSR	3	3	2	
Mergoum et al., 2013 [112]	13	13	SteeleND X ND735 Hard X Hard	RIL	129	1789	SSR, DArT	4	4	4	
Wang et al., 2014 [119]	-		GWAS population. 94 diverse lines with phenotypes of hard, soft and extra soft kernels	GWAS	94	1193 *	SSR, DArT	2	0	0	
Li et al., 2016 [113]	14	14	Ning7840 X Clark Hard X Soft	RIL	127	4225	SSR, SNP	2; 5; ^9^	4	6	Yes
Jernigan et al., 2018 [120]	-	15	GWAS population. including 469 lines with historical phenotypes	GWAS	469	15,229 *	SNP	5	0	5	
Boehm Jr. et al., 2018 [114]	-		Butte86 X ND2603 Hard X Hard	RIL	132	1813	SSR, SNP, Functional markers	4	na	na	
Ibba et al., 2019 [117]	-	16	Creso X Langdon 1-67 8Hard X Soft	RIL	428	8495 **	SNP, TAS, Functional markers	2; 24; ^10^	0	20	
Kumar et al., 2019a [115]	-	17	Alpowa X BC2F5SS163 Soft X Extra Soft	RIL (F3:F5)	125	913	SNP	10	0	8	
Kumar et al., 2019b [116]	15	18	Alpowa X BC2F5SS163 Soft X Extra Soft	RIL (F6)	229	NA	KASP, SSR	4	1	4	
Kumar et al., 2019c [118]	-	19	ND705 X PI414566 Hard X Hard	RIL	160	4676	SNP	7 ^11^	0	7	

^1.^ This column denotes the corresponding track number (Track#) on the consensus genetic map (Figure S1). ^2.^ This column denotes the corresponding track number (Track#) on the reference genome CS RefSeq v1.0 (Figure 1). ^3.^ This column denotes the type of population. In particular, two studies (Kumar et al., 2019a [113] and Kumar et al., 2019b [114]) used the same bi-parental RIL population but different generations, as noted in Table 1. ^4.^ This column denotes population size. For the study by Li et al., 2012 [106], two populations were used: “WJ” stands for the RIL population developed from Weimai 8 x Jimai 20, while “WY” stands for Weimai 8 x Yangnong 9. ^5.^ This column denotes the size of the genetic maps (cM as the unit) when the corresponding study used a linkage mapping approach. In particular, for the two GWAS studies [117,118], the number of markers used for GWAS analysis is given. For the study by Ibba et al., 2019 [115], since most of the GBS-SNP markers (~15,000) are single dosage markers (dominant markers), the number of markers used for single marker–trait association is given. ^6.^ One trait-associated marker (Xgwm130) is unlinked to the genetic map and, therefore, cannot be mapped to the consensus genetic map or the reference genome. ^7.^ Two QTLs were identified for NIRS-based kernel hardness, while four QTLs were identified for SKCS-based kernel hardness. ^8.^ Three QTLs were identified for the Weimai 8 X Jimai20 RIL population, while five QTL were identified for the Weimai 8 X Yangnong19 RIL population. ^9.^ Two QTLs were identified for NIRS-based kernel hardness, while five QTLs were identified for SKCS-based kernel hardness. ^10.^ Two major QTLs were identified; 24 significant SNPs were associated with kernel hardness, forming 18 SNP signals (including single SNPs and SNP clusters). ^11.^ Seven QTLs or trait-linked SNPs were identified, with the QTL regions defined by multiple associated SNPs.

## Supplementary Materials

Supplementary materials can be found at https://www.mdpi.com/2223-7747/9/12/1631/s1. Figure S1: Projection of the QTL regions and genetics markers associated with wheat kernel hardness on the consensus genetic map of common wheat. Figure S2: Size distribution of the QTL reviewed in the present study. Table S1: Detailed information on the QTLs and their associated marker projected on the common wheat reference genome CS RefSeq v1.0. Table S2: Detailed information on the QTLs and their associated markers projected on the common wheat consensus genetic map.

## Abbreviations

AFLP	Amplified fragment length polymorphism
chr.	Chromosome
CRP	Crush response profile
CS	Chinese Spring
DArT	Diversity array technology
DH	Double haploid
GBS	Genotyping-by-sequencing
GSP	Grain Softness Protein
*Ha*	*Hardness*
HI	Hardness Index
HRS	Hard Red Spring
HRW	Hard Red Winter
HW	Hard White
IWGSC	International Wheat Genome Sequencing Consortium
KASP	Kompetitive allele-specific PCR
MAGIC	Multiparent Advanced Generation Inter-Cross
NAM	Nested association mapping
NIRS	Near-infrared spectroscopy
PCR	Polymerase chain reaction
PIN	Puroindoline
PINA	Puroindoline A
PINB	Puroindoline B
QTL	Quantitive trait loci
RFLP	Restriction Fragment Length Polymorphism
RIL	Recombinant inbred lines
SNP	Single-Nucleotide Polymorphism
STS	Sequence-Tagged Sites
SKCS	Single Kernel Characterization System
SSR	Simple Sequence Repeats
SRW	Soft Red Winter
SW	Soft White
USDA	United States Department of Agriculture
