# Supplementary material for: Toward the Genetic Basis and Multiple QTLs of Kernel Hardness in Wheat
Source: Plants (Basel). 2020 Nov 24;9(12):1631. doi: 10.3390/plants9121631 (PMC7760206; doi:10.3390/plants9121631)
Supplement: Supplementary file 1 [file plants-09-01631-s001.zip › Yin_Plants_SuppFigure.docx]

Perspective

*Toward the Genetic Basis and Multiple QTL Loci of Wheat Kernel Hardness*

**Min Tu^1^, Yin Li ^1,*^**

^1^ Waksman Institute of Microbiology, Rutgers, the State University of New Jersey, 190 Frelinghuysen Road, Piscataway, NJ 08854, USA.

***** Correspondence: liyin.plant@hotmail.com, yl737@waksman.rutgers.edu (Y.L.); Tel.: +01-848-445-6446 (Y.L.)

**Supplementary Materials**

**
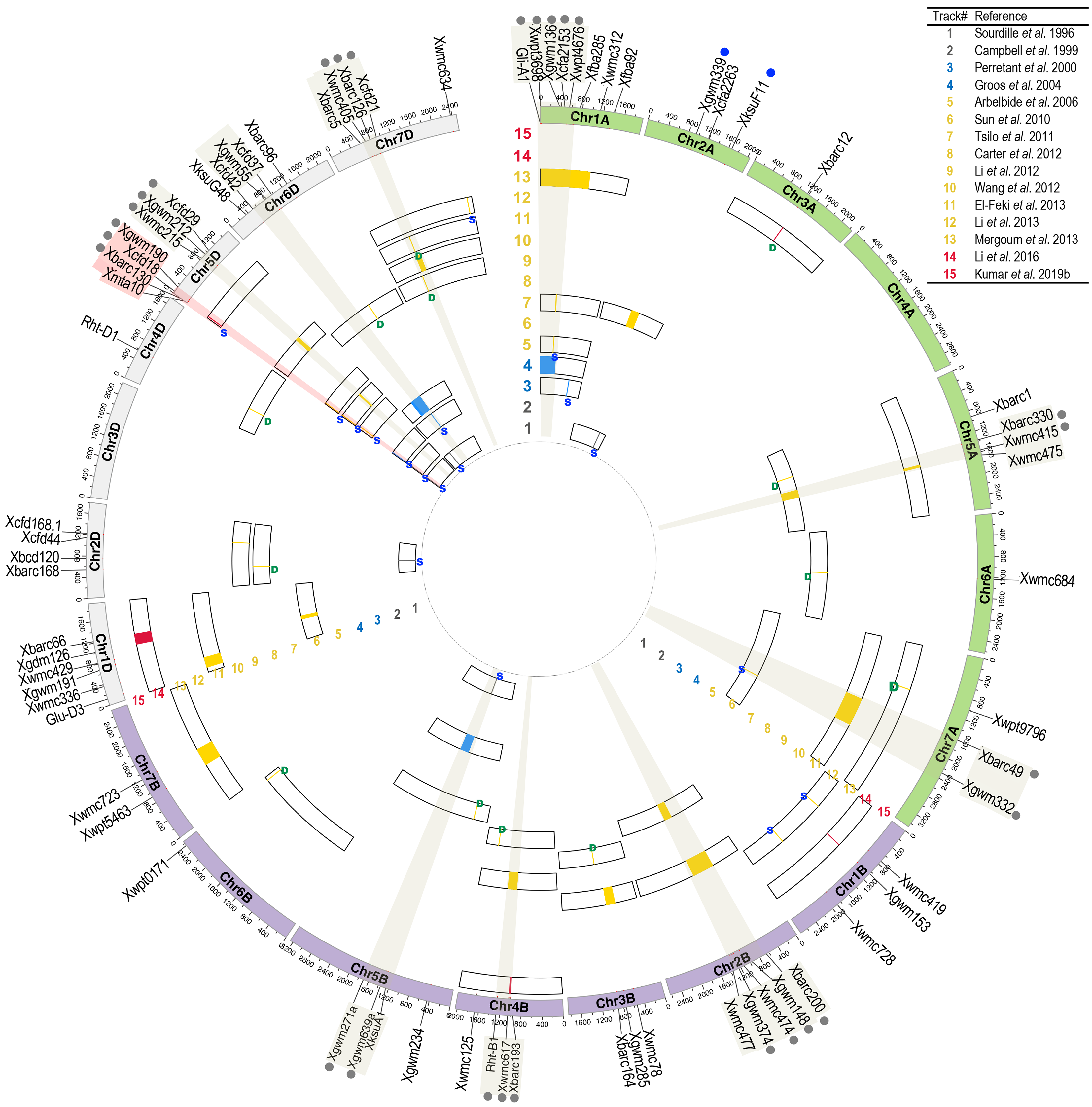
**

**Figure S1.** **Projection of the QTL regions and genetics markers associated with wheat kernel hardness on the consensus genetic map of common wheat**. The QTL regions associated with kernel hardness were retrieved from the fifteen QTL-mapping studies. The detailed information about these QTL-mapping studies and their projected QTL regions or markers are provided in Table 1 and Supplementary Table S2. The left and right boundary of a QTL were defiend by two genetic markers according to the study where it came from. In some cases, only one of the left or right marker could be accurately located on the consensus genetic map. When both of the left and right boundary markers can be located on the genetic map, the QTL region is indicated using green letter “D” on the figure (meaning “double markers”). When only one of the left or right boundary marker can be located on the consensus map, the QTL region is indicated using blue letter “S” (meaning “single marker”). The out-most track denotes the consensus genetic map of common wheat with centiMorgans labeled. The A, B, D genomes are colored in green, purple and grey, respectively. The tracks from outer to inner represent the fifteen QTL mapping studies, labeled using the first author and the year of publication. For each study (track), only the linkage group (chromosome) which a kernel hardness-associated QTL region or marker could be located on are shown, with the QTL regions or markers highlighted in colors (i.e., grey, blue, yellow and red). The colors used to highlight the QTL regions, grey, blue, yellow and red, denote the majority type of markers used in each QTL mapping study as RFLP, RFLP + SSR, SSR + DarT, and SNP, respectively. All of the markers used for QTL/marker projection are labeled outside the wheat genome track.


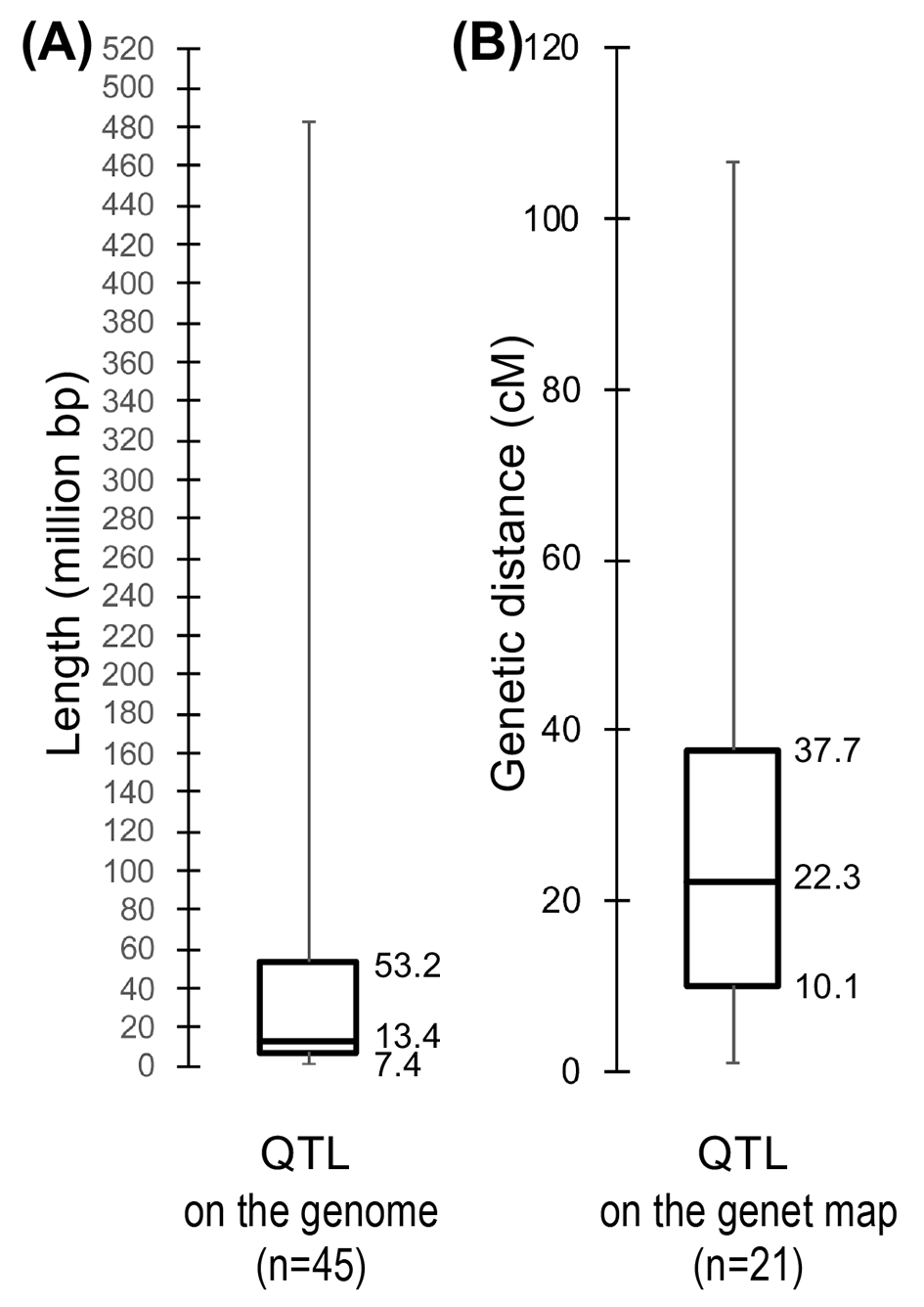


**Figure S2. Size distribution of the QTL reviewed in the present study.**

The QTL reviewed herein have several different situations: (1) a QTL of which both the left and right assocaited markers are able to be projected on the genome or the genetic map (21 and 45 such type of QTL projected on the genetic map and the reference genome, respectively); (2) a QTL of which only one of the two associated markers are able to projected on the genome or the genetic map (15 and 11 such type of QTL projected on the genetic map and the reference genome, respectively); (3) a QTL with only one associated marker reported; (4) a single trait-associated marker was identified in GWAS analysis or single marker-association analysis. Only the type-1 projected QTL were used for calculating QTL size distribution when projected onto the reference genome (A) and the consensus genetic map (B), with the first quartile, median and third quartile for QTL size distribution labled on the figures. A total ninety-nine QTL have been projected on the bread wheat reference genome, while fifty-four QTL have been projected on the consensus genetic map.

| 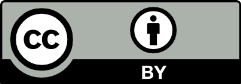 | © 2020 by the authors. Submitted for possible open access publication under the terms and conditions of the Creative Commons Attribution (CC BY) license (http://creativecommons.org/licenses/by/4.0/). |
| --- | --- |
